# Comparing development of synaptic proteins in rat visual, somatosensory, and frontal cortex

**DOI:** 10.3389/fncir.2013.00097

**Published:** 2013-05-28

**Authors:** Joshua G. A. Pinto, David G. Jones, Kathryn M. Murphy

**Affiliations:** ^1^McMaster Integrative Neuroscience Discovery and Study Program, McMaster UniversityHamilton, ON, Canada; ^2^Pairwise Affinity Inc.,Dundas, ON, Canada; ^3^Psychology, Neuroscience and Behavior, McMaster UniversityHamilton, ON, Canada

**Keywords:** synapsin, synaptophysin, PSD-95, gephyrin, critical period, integrated network, E-I balance, cortical development

## Abstract

Two theories have influenced our understanding of cortical development: the integrated network theory, where synaptic development is coordinated across areas; and the cascade theory, where the cortex develops in a wave-like manner from sensory to non-sensory areas. These different views on cortical development raise challenges for current studies aimed at comparing detailed maturation of the connectome among cortical areas. We have taken a different approach to compare synaptic development in rat visual, somatosensory, and frontal cortex by measuring expression of pre-synaptic (synapsin and synaptophysin) proteins that regulate vesicle cycling, and post-synaptic density (PSD-95 and Gephyrin) proteins that anchor excitatory or inhibitory (E-I) receptors. We also compared development of the balances between the pairs of pre- or post-synaptic proteins, and the overall pre- to post-synaptic balance, to address functional maturation and emergence of the E-I balance. We found that development of the individual proteins and the post-synaptic index overlapped among the three cortical areas, but the pre-synaptic index matured later in frontal cortex. Finally, we applied a neuroinformatics approach using principal component analysis and found that three components captured development of the synaptic proteins. The first component accounted for 64% of the variance in protein expression and reflected total protein expression, which overlapped among the three cortical areas. The second component was gephyrin and the E-I balance, it emerged as sequential waves starting in somatosensory, then frontal, and finally visual cortex. The third component was the balance between pre- and post-synaptic proteins, and this followed a different developmental trajectory in somatosensory cortex. Together, these results give the most support to an integrated network of synaptic development, but also highlight more complex patterns of development that vary in timing and end point among the cortical areas.

## INTRODUCTION

During the critical period, substantial changes in expression of pre- and post-synaptic proteins interact with experience to facilitate synaptic development, fine tuning of cortical circuits, and maturation of the cortical connectome. The nascent synaptic proteome provides the ready plasticity that underlies functional development of synapses and cortical circuits. Two influential theories have been proposed to describe how synaptic development proceeds across the cortex: the integrated network and the cascade of development. An integrated network of synaptic development, with cortical areas developing in unison, was proposed when autoradiography of a collection of post-synaptic receptors found common developmental trajectories in different cortical areas (Lidow et al., 1991). In contrast, a cascade of synaptic development, with a system-by-system wave of maturation, was proposed when electron microscopy counts of synapses showed changes starting in primary sensory areas and proceeding to higher order cortical areas (Huttenlocher, 1990; Huttenlocher and Dabholkar, 1997). Those anatomical studies present opposing views on cortical development that raise challenges for current work aimed at comparing detailed maturation of the connectome among cortical areas. We have taken a different approach and quantified the available pool of a small set of pre- and post-synaptic proteins in sensory and higher order cortical areas to capture developmental changes across the cortex for these building blocks of synapses.

There are nearly 3000 synaptic proteins (Filiou et al., 2010) that provide robust diversity and determine the function of individual synapses. The pre-synaptic proteome has hundreds of proteins (Bayes and Grant, 2009), many of which are directly involved with the cycling of synaptic vesicles. Synapsin and Synaptophysin are the most abundant synaptic vesicle proteins and have distinct functional roles. They are also the most commonly used markers for pre-synaptic terminals (Micheva et al., 2010), which points to them as good candidates to capture pre-synaptic development. Synapsin is the most specific marker for pre-synaptic terminals (Micheva et al., 2010); it is the major peripheral membrane protein accounting for 6% of the total synaptic vesicle protein (Huttner et al., 1983), regulating the reserve pool of synaptic vesicles available for exocytosis (Bahler et al., 1990), and maintaining the organization and abundance of vesicles at pre-synaptic terminals (Bykhovskaia, 2011). Synaptophysin is the major integral membrane protein accounting for 6–10% of total synaptic vesicle protein (Jahn et al., 1985; Wiedenmann and Franke, 1985; Takamori et al., 2006), regulating the kinetics of synaptic vesicle endocytosis (Kwon and Chapman, 2011), and synaptic vesicle retrieval through its interaction with synaptobrevin (Gordon et al., 2011). Synapsin is found at all glutamatergic and GABAergic synaptic boutons, while Synaptophysin levels are highest at glutamatergic and very low at GABAergic terminals (Gronborg et al., 2010; Micheva et al., 2010). Importantly, the development of Synapsin and Synaptophysin expression is required for the maturation of pre-synaptic function and stabilization of pre-synaptic boutons (Hopf et al., 2002). Furthermore, the balance between these proteins will effect vesicle cycling and likely impact the probability of transmitter release, especially after strong or sustained stimulation.

The post-synaptic proteome contains about 2,000 proteins (Collins et al., 2006; Trinidad et al., 2008) and in the cortex it is dominated by proteins for glutamatergic (GluA, GluN) and GABAergic (GABA_A_) receptors. There is tremendous diversity in the subunit makeup of these classes of receptors, and the complexity is increased by the many intracellular components for receptor trafficking, functioning, and signaling. GluA receptors have 4 subunit types (Jackson and Nicoll, 2011), GluN receptors have 3 subunit types, GluNR1, GluNR2, and GluNR3, with multiple variants and distinct isoforms (Jackson and Nicoll, 2011), and GABA_A_R have 19 subunit types (Sieghart, 1995; Olsen and Sieghart, 2009). This post-synaptic complexity is reduced, however, at the level of scaffolding proteins where post-synaptic density-95 (PSD-95) anchors GluA and GluN receptors, and Gephyrin anchors GABA_A_ receptors. Furthermore, PSD-95 and gephyrin are the most commonly used markers for excitatory and inhibitory synapses, which points to them as good markers to capture post-synaptic development.

Post-synaptic density-95 is required for functional GluA and GluN containing synapses, and when knocked-out, there is an increase in the number of silent synapses (Beique et al., 2006). Gephyrin knock-down decreases the number of GABA_A_ receptor clusters, showing that it is required for stabilization of GABAergic synapses (Yu et al., 2007). Both PSD-95 and Gephyrin are motile among a localized group of synapses (Gray et al., 2006; Dobie and Craig, 2011), with the total amount of PSD-95 or gephyrin limiting the number of synapses (Keith and El-Husseini, 2008). At any given time, however, only a fraction of the protein is located at the synapse. In addition, interactions between PSD-95 and gephyrin regulate the number of excitatory and inhibitory synapses and affect the physiological excitatory or inhibitory (E-I) balance (Prange et al., 2004; Lardi-Studler et al., 2007; Keith and El-Husseini, 2008). The E-I balance shifts during early development (Dorrn et al., 2010), and it is important for triggering the start of the critical period for ocular dominance plasticity in visual cortex (Hensch and Fagiolini, 2005). Taken together, these attributes point to PSD-95 and Gephyrin as good post-synaptic markers to capture development of excitatory and inhibitory synapses and the E-I balance.

Although there is remarkable diversity in the synaptic proteome, quantifying just a few markers, the pre-synaptic proteins synapsin and synaptophysin, plus the post-synaptic proteins PSD-95 and gephyrin, can capture multiple aspects of synaptic development including developmental expression of each synaptic protein, the balance among all proteins, and the excitatory or inhibitory (E-I) balance. These are important measures for comparing synaptic development among cortical areas and we set out to study development of visual, somatosensory, and frontal cortex by quantifying the available pool of these four synaptic proteins. We found that development of the individual proteins and the post-synaptic index overlapped among the three cortical areas, but the pre-synaptic index developed later in frontal cortex. We applied a neuroinformatics approach using principal component analysis (PCA) to characterize the multidimensional changes during development of these synaptic proteins. Developmental increases in the total protein expression were similar among the cortical areas, but the E-I balance emerged as waves, and the balance between pre- and post-synaptic proteins followed a different trajectory in somatosensory cortex. Together, these results give the most support for an integrated network of development, but also highlight more complex patterns of development that vary in timing and end point among the cortical areas.

## MATERIALS AND METHODS

### TISSUE SAMPLE COLLECTION

A total of 40 tissue samples were collected from primary visual (A/P -5 to -8 mm, M/L 2–4 mm, *n* = 13), frontal (A/P 0–2 mm, M/L 2–5 mm, *n* = 14), and somatosensory cortex (A/P -4 to -2 mm, M/L 2–5 mm, *n* = 13) from long evans rats ranging in age from 0 to 93 days (visual, range = P4–P93; frontal, range = P0–P93; somatosensory, range = P0–P74; **Table 1**). The rats were euthanized with Euthanol (150 mg/kg) and perfused transcardially with cold 0.1 M PBS (4°C) until circulating fluid was cleared. The brain was quickly removed and immersed in cold PBS, a sample was cut out from each of the cortical regions (approximately 3 mm × 2 mm, A/P coordinates relative to Bregma) then quickly frozen on dry ice and stored at -80°C. Tissue samples from visual cortex were collected at P0, however, they did not contain enough synaptic protein to be measured by Western blotting.

**Table 1:** Cortical tissue samples.

**Postnatal Age (Days)**	**Visual**	**Somatosensory**	**Frontal**
0		x	x
4	x	x	x
7		x	
11	x	x	x
14	x	x	x
17	x	x	x
21	x	x	x
25	x	x	x
28	x	x	x
31	x	x	x
35	x	x	x
45	x	x	x
64	x		x
74	x	x	x
93	x		x

All experimental procedures were approved by the McMaster University Animal Research Ethics Board.

### TISSUE SAMPLE PREPARATION

To quantify the available pool of synaptic proteins, tissue samples (50–100 mg) were suspended in cold homogenization buffer (1 ml buffer:50 mg tissue – 0.5 mM DTT, 1mM EDTA, 2 mM EGTA, 10 mM HEPES, 10 mg/L leupeptin, 100 nM microcystin, 0.1 mM PMSF, 50 mg/L soybean trypsin inhibitor) and homogenized in a glass-glass Dounce homogenizer (Kontes, Vineland, NJ, USA). The homogenized sample was removed and added to 10% sodium-dodecylsulfate (SDS). Protein concentrations were determined using the bicinchoninic acid (BCA) assay guidelines (Pierce, Rockford, IL, USA). A control sample was made by combining a small amount of the prepared tissue sample from each of the 40 samples.

For this study we used whole homogenate samples and not synaptoneurosomes to quantify the total pool of the synaptic proteins. We chose to use the homogenate for two reasons: first, the proteins we quantified have high abundances; second, during the first 7 days of postnatal development in the rat there is very low protein yield in the cortex, significant numbers of mature cortical synapses do not emerge until about P7 (Li et al., 2010) and neurons are still migrating, differentiating, and forming cortical layers early in development until about P20 (Hicks and D’Amato, 1968). These immaturities, especially the low protein yield, mean that the filtration and centrifugation steps used to concentrate synaptic proteins in the synaptoneurosome preparation will lead to different levels of synaptic enrichment in neonatal versus older tissue (Balsor and Murphy, Unpublished Observation).

### IMMUNOBLOTTING

The homogenate samples (25 μg) were separated on SDS–polyacrylamide gel electrophoresis (SDS–PAGE) mini-gels (Precise Protein Gels, Pierce Biotechnology Inc., Rockford, IL, USA) and transferred to polyvinylidene difluoride (PVDF-FL) membranes (Millipore, Billerica, MA, USA). Each sample was run two or three times. Blots were pre-incubated in blocking buffer (Odyssey Blocking Buffer 1:1 with PBS) for 1 hour (Li-cor Biosciences; Lincoln, NE, USA), then incubated in primary antibody overnight at 4°C using the following concentrations: synapsin 1a/b (80–77 kDa), 1:8000 (Invitrogen, Carlsbad, CA, USA); synaptophysin (38 kDa), 1:2000 (Sigma-Aldrich, St. Louis, MO, USA); PSD-95 (95 kDa), 1:32000 (Millipore, Billerica, MA, USA); Gephyrin (93 kDa), 1:2000 (Millipore, Billerica, MA, USA); GAPDH (37 kDa), 1:4000 (Imgenex, San Diego, CA, USA). The blots were washed with PBS containing 0.05% Tween (Sigma, St. Louis, MO, USA) (PBS-T; 3 × 10 min), incubated for 1 hour at room temperature with the appropriate IRDye labeled secondary antibody, (Anti-Mouse, 1:8000, Anti-Rabbit, 1:10,000; Li-cor Biosciences; Lincoln, NE, USA), and washed in PBS-T (3 × 10 min). The bands were visualized using the Odyssey scanner (Li-cor Biosciences; Lincoln, NE, USA). The combination of the IRDye secondary antibodies and Odyssey scanner system provides a wide linear dynamic range so that both strong and weak bands could be accurately quantified on the same blot. The blots were stripped and prepared to be re-probed with additional antibodies (Blot Restore Membrane Rejuvenation kit, Chemicon International, Temecula, CA, USA). We determined that both the amount of protein loaded in each well and the concentration of each antibody was within that linear range.

### ANALYSIS

To analyze the bands, we scanned the blots (Odyssey infrared scanner) and quantified the bands using densitometry (Licor Odyssey Software version 3.0; Li-cor Biosciences; Lincoln, NE, USA). The Odyssey system uses near infrared-dyes for antibody detection, providing a 16–250-fold wider linear range than chemiluminescence (Schutz-Geschwender et al., 2004). Density profiles were determined by performing a subtraction of the background, integrating the pixel intensity across the area of the band, and dividing the intensity by the width of the band to control for variations in lane width. GAPDH normalization was used as the loading control and for each sample the expression of the synaptic protein was divided by GAPDH expression. The control sample (a mixture of all the samples) was run on all of the gels and the density of each sample was measured relative to that control (sample density/control density).

The developmental trajectories for each of the four proteins in the three cortical areas were visualized by plotting the expression levels for all samples (light colored dots) as well as the average expression (dark colored dots) for each animal. Many of the developmental changes followed a monotonic increase or decrease and we used a model fitting approach (Christopoulos and Lew, 2000) to fit curves that allowed us to describe and compare these developmental trajectories. Using the online tool ZunZun.com an exponential decay function [y = y_o_+a*exp(-t/τ); solid lines], where *t* = age in days, was fit to the results from all samples. The best fitting curve was found by least squares and the goodness of fit (R), and the statistical significance of the fit (p) were determined. The time constant (τ) for the rise or fall of expression level was calculated from the exponential decay function. We defined the age when adult level was reached as 3τ. This provided an objective measure representing the age when protein expression reached 87.5% of the asymptotic level. In addition, we calculated 95% confidence intervals (CI) around the decay functions (dotted lines). To determine the upper and lower 95% confidence limits we assumed the data for each individual animal was normally distributed. We multiplied the standard error of the mean for each animal by 1.96 (0.975 quantile of the normal distribution) then added or subtracted that value from the mean of each individual animal providing us with upper and lower 95% confidence limits for each animal. We then fit exponential decay functions to the upper and lower limits of the 95% CIs to visualize the predicted range of protein expression at any given age. Gephyrin had a different developmental trajectory with a brief peak when there was rapid change in expression. This type of brief increase in inhibitory synapses has been found previously in electronmicroscopy counts (Blue and Parnavelas, 1983) so we fit a peak function [y = a*exp(-0.5*(ln(t/b)/c)^2^)] to the gephyrin expression. The peak parameter (t) provided a measure of the timing of maximum gephyrin expression for all three cortical areas. To determine if the timing of development for the four proteins was significantly different among the three cortical areas, we performed a two-sample *z*-tests using 3τ or the peak parameter (t) and their respective standard errors.

We quantified the relationship between pre- and post-synaptic proteins by calculating two indices that measured the developmental differences between the pairs of pre-synaptic (synapsin and synaptophysin) or post-synaptic proteins (PSD-95 and gephyrin). The indices provide an indication of synaptic development because each pair of proteins is functionally related: synapsin and synaptophysin expression is required for pre-synaptic function and stabilization of pre-synaptic boutons (Hopf et al., 2002); interactions between PSD-95 and gephyrin regulate the number of excitatory and inhibitory synapses and affect the physiological E/I balance (Prange et al., 2004; Lardi-Studler et al., 2007; Keith and El-Husseini, 2008). In addition, this type of contrast index is common approach in signal processing to determine the quality of the signal and here provided an analysis of pre- or post-synaptic development. Pre-synaptic index – [(synapsin-synaptophysin)/(synapsin+synaptophysin)], post-synaptic index – [(PSD-95-gephyrin)/(PSD-95+gephyrin)]. An exponential decay function was fit to the pre-synaptic index, while a sigmoid function was fit to the post-synaptic index [y = a/(1.0+exp(-(x–t)/b))+c]. For the sigmoid function, adult levels represented the age when the post-synaptic index reached 87.5% of the asymptotic level. 95% CIs calculated and plotted as described above, and two sample *z*-tests were performed to quantify significant differences among the three cortical areas.

### PRINCIPAL COMPONENT ANALYSIS

A multivariate analysis of the expression pattern for all proteins was done using PCA. Protein expression was compiled into an *mxn* matrix. The rows (*m* = 4) represent the proteins (synapsin, synaptophysin, PSD-95, gephyrin), and the columns (*n* = 40) represent protein expression levels for each of the 40 samples (Visual = 13, Somatosensory = 13, Frontal = 14). The data were centered by subtracting the mean column vector, and then a singular value decomposition (SVD) was applied to calculate the principal components in Matlab (The Mathworks, Inc., Natick, MA, USA). The SVD represents the expression level for all proteins from one sample as a vector in high dimensional space. The PCA identifies the directions in “protein expression space” that represent the variance in the data across all cortical areas.

In this study, there were four principal components; the first three accounted for 99% of the variance in the data. A commonly used rule of thumb is that principal components accounting for up to 80% of the cumulative variance are considered significant. To be more precise, the data were analyzed using a bootstrapping method. We performed a Monte Carlo simulation with 100,000 repetitions. For each, the simulated data set had the same number of rows (proteins) and columns (samples), and the simulated protein expression levels were drawn randomly from a normal distribution with the same mean and standard deviation as the original data. For each iteration, a PCA was performed and we calculated how much of the residual variance each of the four principal components accounted for. Our experimental principal components were deemed to be statistically significant if they accounted for a much greater proportion of the residual variance than would be expected from random simulation. For example, a principal component was significant with *p* < 0.05 if it accounted for more of the residual variance than was observed in 95% of the simulated iterations. To determine biological links and to aid interpretation of the significant principal components, we used an approach that we developed (Beston et al., 2010) and calculated correlations between each significant principal component and several biologically relevant measures. These included: expression levels of the four proteins, the pre- and post-synaptic index, total protein expression, and a pre-synaptic:post-synaptic index. The significance level for identifying potential biological correlates was adjusted to *p* < 0.0021 using the Bonferroni correction for multiple comparisons. The PCA results were visualized using scatterplots, with PCA coordinates on the *y*-axis, and age on the *x*-axis. We fit exponential decay and peak functions where appropriate, as well as a logistics functions [y = a + b/(1+exp(0.5*(x-t)))] when there was an abrupt monotonic decrease in expression. The inflection point parameter (t) provides an objective measure of the timing of the abrupt decrease in expression. We performed two-sample *z*-tests were performed to determine if there were any significant differences in the timing of development among the three cortical areas. To determine if there were significant differences in expression levels of principal component 3, we used an analysis of variance, and a Tukeys *post-hoc* test.

Quantification of total protein expression and a pre-synaptic:post-synaptic index was done after PCA. Total protein expression was calculated by summing the expression of the four proteins for each sample. Total protein expression was the visualized using a scatterplot, with total protein expression on the *y*-axis, and age on the *x*-axis. We fit exponential decay functions to total protein expression for each cortical area, as previously described, and performed two-sample *z*-tests to determine if there were significant differences in the timing of development among the three areas. The pre-synaptic:post-synaptic (pre:post) index [(synapsin+synaptophysin)-(PSD-95+gephyrin)/(synapsin+synaptophysin+PSD-95+gephyrin)] was calculated for each sample and then visualized using a scatterplot, with the pre:post index on the *y*-axis, and age on the *x*-axis. We fit exponential decay functions to the pre:post index for visual and frontal cortex, and performed a two-sample *z*-test to determine if there was a significant difference in the timing of development between the areas. We fit a weighted average curve to the pre:post index for somatosensory cortex.

## RESULTS

### GAPDH LOADING CONTROL DURING CORTICAL DEVELOPMENT

**FIGURE 1:**
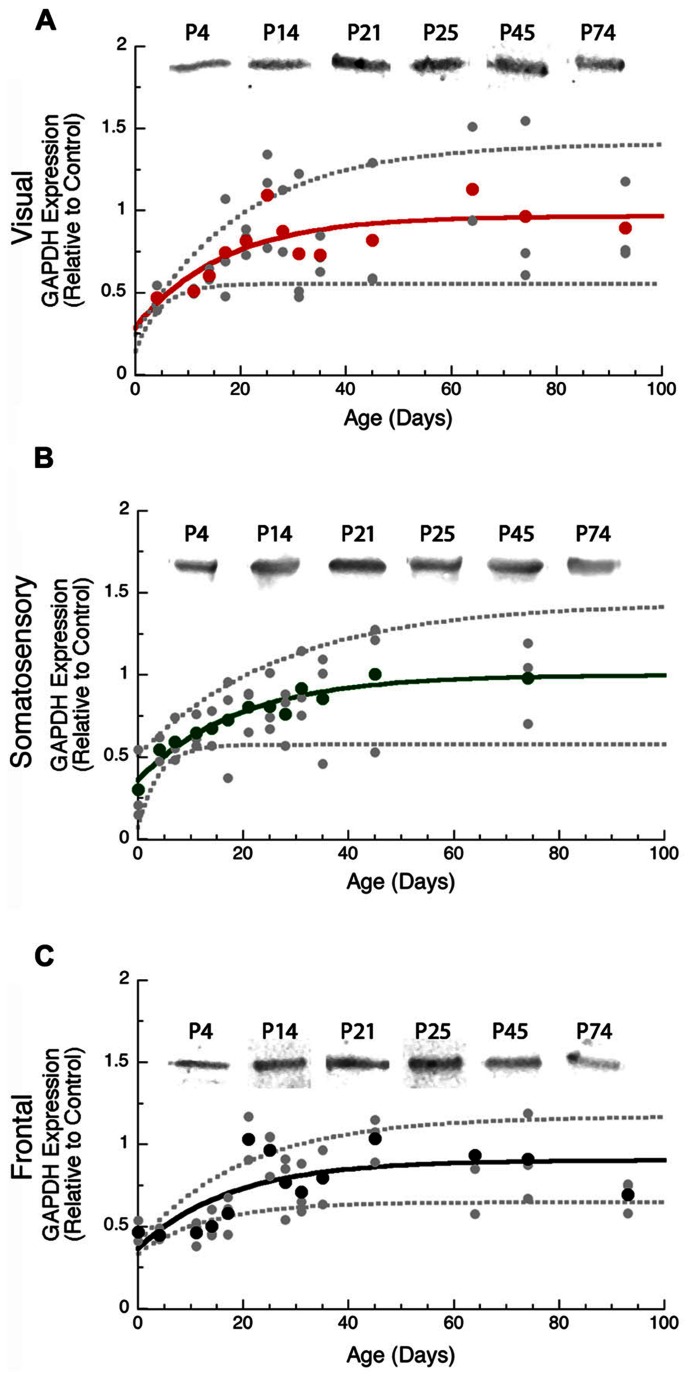
**Developmental changes in GAPDH in rat visual (red, A), somatosensory (green, B), and frontal (black, C) cortex.** Gray dots are results from all runs, and color dots are the average for each animal. Example bands are shown above the graphs. Exponential decay functions were fit (solid lines), and 95% confidence intervals (dotted lines) were added, adult levels are defined as 3τ. GAPDH reached adult levels in visual **(A)** at P50 (3τ = P49.9 ± 30.4; curve-fit *R* = 0.50; *p* < 0.001), somato - sensory **(B)** at P57 (3τ = P57.1 ± 26.9; curve-fit *R* = 0.69; *p* < 0.0001), and frontal** (C)** at P53 (3τ = P52.6 ± 24.3; curve-fit *R* = 0.69; *p* < 0.0001).

We used GAPDH expression as the loading control for this study and examined development of GAPDH expression in the cortex. During the first 14–20 days of postnatal development neurons are still migrating into the rat cortical plate, differentiating, and forming the cortical layers (Hicks and D’Amato, 1968) and we found that GAPDH expression also increased over that time period (**Figure 1**). Thus, it was important to use GAPDH expression as the control for non-specific protein levels so that subsequent quantification of pre- and post-synaptic proteins reflected specific development of the available pool of synaptic proteins rather than a non-specific increase in total cortical proteins.

### PRE-SYNAPTIC DEVELOPMENT IS LED BY SYNAPSIN AND HAPPENS FIRST IN SOMATOSENSORY CORTEX

To examine pre-synaptic development we quantified the expression of pre-synaptic proteins, synapsin and synaptophysin, in rat visual, somatosensory, and frontal cortex. Both proteins are components of the synaptic vesicle membrane and are involved in organizing vesicles at pre-synaptic terminals (Bykhovskaia, 2011), but they have different functions: synapsin regulates the pool of vesicles for exocytosis (Bahler et al., 1990); and synaptophysin regulates the kinetics of vesicle endocytosis (Kwon and Chapman, 2011). In addition, synapsin is the most specific pre-synaptic maker (Micheva et al., 2010). Together, the expression of synapsin and synaptophysin provides information about both function and number of pre-synaptic terminals.

**FIGURE 2:**
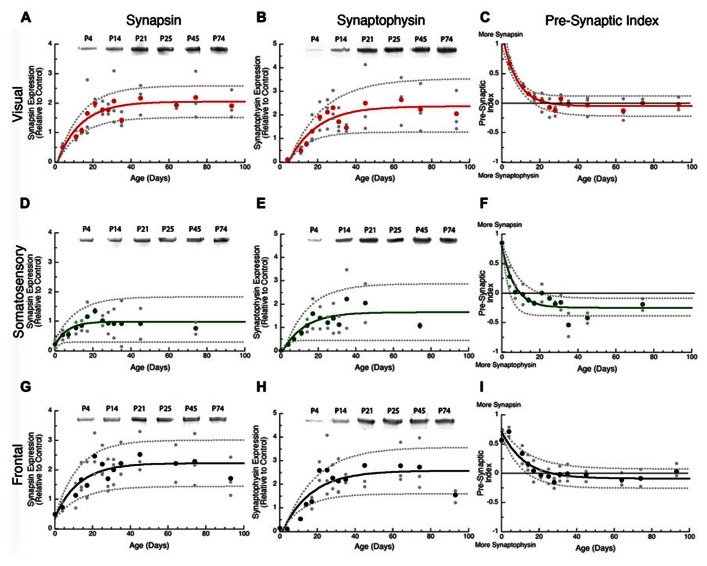
**Developmental changes in synapsin (A,D,G), synaptophysin (B,E,H), and the pre-synaptic index (C,F,I) in rat visual (red), somatosensory (green) and frontal (black) cortex.** Gray dots are results from all runs, and color dots are the average for each animal. Example bands for each protein and area are shown above the graphs. Exponential decay functions were fit (solid lines), and 95% confidence intervals (dotted lines) were added, adult levels are defined a 3τ. Synapsin reached adult levels in visual **(A)** at P37 (3τ = P37.2 ± 12.6; curve-fit *R* = 0.70; *p* < 0.0001), somatosensory **(D)** at P19 (3τ = P19.0 ± 14.9; curve-fit *R* = 0.45; *p* < 0.005), and frontal **(G)** at P38 (3τ = P37.6 ± 13.5; curve-fit *R* = 0.70; *p* < 0.0001). Synaptophysin reached adult levels in visual **(B)** at P45 (3τ = P44.8 ± 15.1; curve-fit *R* = 0.72; *p* < 0.0001), somatosensory **(E)** at P35 (3τ = P34.7 ± 22.2; curve-fit *R* = 0.64; *p* < 0.0001), and frontal **(H)** at P45 (3τ = P45.4 ± 14.2; curve-fit *R* = 0.75; *p* < 0.0001). Pre-synaptic index reached adult levels in visual **(C)** at P24 (3τ = P23.5 ± 4.2; curve-fit *R* = 0.88; *p* < 0.0001), somatosensory **(F)** at P20 (3τ = P20.2 ± 6.0; curve-fit *R* = 0.82; *p* < 0.0001), and frontal **(I)** at P37 (3τ = P36.8 ± 7.8; curve-fit *R* = 0.85; *p* < 0.0001).

In all three cortical areas, we found that synapsin developed before synaptophysin (**Figure 2** versus **Figure 4A**) and all of the changes were well fit by τ decay functions. Initially, synapsin expression was low and then rose rapidly to reach adult levels. Somatosensory cortex developed first, increasing two-fold to reach adult levels at P19 (**Figure 2D**, curve-fit *R* = 0.45; *p* < 0.005, 3τ = P19.0 ± 14.9). Synapsin expression increased about threefold in visual and frontal cortex to reach adult levels at P37 and P38, respectively (visual **Figure 2A**, curve-fit *R* = 0.70; *p* < 0.0001, 3τ = P37.2 ± 12.6; frontal **Figure 2G**, curve-fit R0.70; *p* < 0.0001, 3τ = P37.6 ± 13.5). To compare the ages when synapsin reached adult levels (3τ) among the three cortical areas we plotted the 3τ ages and their standard errors (**Figure 4**) and ran a *z*-test to determine if there were any significant differences. We found a lot of overlap around the ages when adult-levels of synapsin were reached and no significant (*z*-test) differences among the three cortical areas.

During the first few days of postnatal development (P0-P4) there was very little expression of synaptophysin in any of the three cortical areas, but then it increased substantially. Synaptophysin reached adult levels (3τ) first in somatosensory cortex, increasing threefold (**Figure 2E**, curve-fit *R* = 0.64 *p* < 0.0001) by P35 (3τ = P34.7 ± 22.2). Between P11 and P45 synaptophysin increased fourfold in visual cortex, and fivefold in frontal cortex when adult levels were reached (**Figure 2B**, curve-fit *R* = 0.72; *p* < 0.0001; 3τ = P44.8 ± 15.1; **Figure 2H**, curve-fit R0.75; *p* < 0.0001, 3τ = P45.4 ± 14.2). There was substantial overlap around the age when adult levels (3τ) were reached in the three areas with no significant differences among the areas (*z*-test, n.s; **Figure 4A**).

### THE SYNAPSIN:SYNAPTOPHYSIN BALANCE IS REACHED LATER IN FRONTAL CORTEX

The overlap among visual, somatosensory, and frontal cortex for the ages when either synapsin or synaptophysin expression reached adult-levels suggests that these pre-synaptic proteins develop at a similar rate in the three cortical areas. It is important to recall that the development of pre-synaptic function depends on both proteins (Hopf et al., 2002), and will be effected by the balance between proteins since they regulate different aspects of synaptic vesicle cycling (exo-cytosis, synapsin endo-cytosis, synaptophysin). To address the balance we calculated an index of synapsin and synaptophysin expression that had a value of +1 when only synapsin was expressed, 0 when there was equal expression, and -1 when only synaptophysin was expressed. We found rapid developmental shifts in this index from substantially more Synapsin to either balanced expression or slightly more synaptophysin that suggests a progressive development from dominance by vesicle exo-cytosis until reaching the adult balance of exo- and endo-cytosis regulation of synaptic vesicle cycling. In visual cortex, the pre-synaptic balance shifted to roughly equal expression reaching adult levels at P24 (**Figure 2C**, curve-fit *R* = 0.88; *p* < 0.0001, 3τ = P23.5 ± 4.2). In somatosensory cortex, the shift was to slightly more synaptophysin by P20 (**Figure 2F**, curve-fit *R* = 0.82; *p* < 0.0001, 3τ = P20.2 ± 6.0). In frontal cortex, the shift was slower and adult levels were not reached until P37 (**Figure 2I**, curve-fit *R* = 0.85; *p* < 0.0001, 3τ = P36.8 ± 7.8). The pre-synaptic balance developed later in frontal than somatosensory cortex (*z*-test, *p* < 0.05), and there was a trend toward frontal developing later than visual cortex (*z*-test, *p* = 0.06; **Figure 4B**). Together, these point to slower development of pre-synaptic function in frontal cortex.

### PSD-95 EXPRESSION INCREASES PROGRESSIVELY BUT GEPHYRIN HAS A BRIEF PERIOD OF RAPIDLY CHANGING EXPRESSION

Both glutamatergic and GABAergic mechanisms are involved in developmental synaptic plasticity, and the physiological E-I balance is central to initiation of the critical period in visual cortex. We examined post-synaptic development of glutamatergic and GABAergic systems by quantifying expression of two proteins – PSD-95 and gephyrin – that anchor excitatory glutamatergic and inhibitory GABAergic receptors, respectively. PSD-95 anchors the excitatory receptors GluA and GluN, and is required for functional GluA and GluN receptors (Beique et al., 2006). Gephyrin anchors the inhibitory GABA_A_ receptor, and is required for the stabilization of GABAergic synapses (Yu et al., 2007). Together, the expression of PSD-95 and gephyrin can provide information about the development of excitatory and inhibitory synapses (Keith and El-Husseini, 2008) especially the E/I balance that is crucial for ocular dominance plasticity in visual cortex (Hensch and Fagiolini, 2005).

**FIGURE 3:**
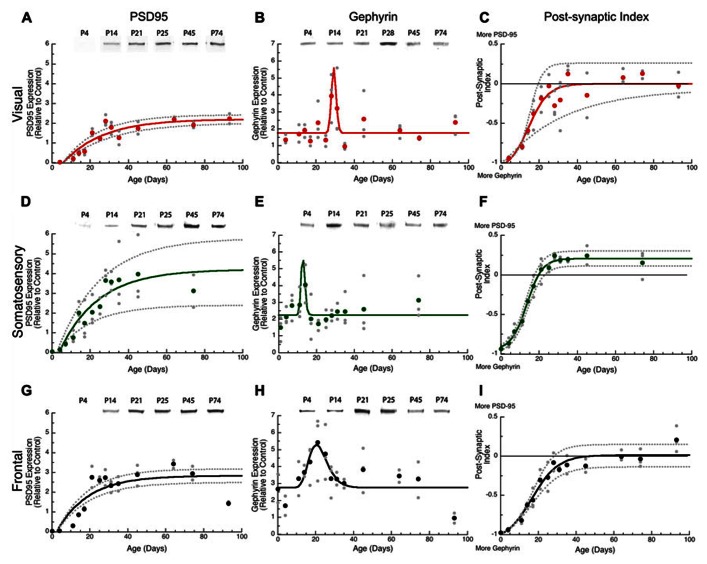
**Developmental changes in PSD-95 (A,D,G), gephyrin (B,E,H), and the post-synaptic index (C,F,I) in rat visual (red), somatosensory (green) and frontal (black) cortex.** Gray dots are results from all runs, and color dots are the average for each. Example bands for each protein and area are shown above the graphs. An exponential decay function was fit (solid line) to PSD-95, a sigmoid function (solid line) was fit to the post-synaptic index, and 95% confidence intervals (dotted lines) were added. Adult levels are defined as 3τ, or the age when 87.5% of the asymptotic expression level was reached. Peak function was fit to gephyrin expression. PSD-95 reached adult levels in visual **(A)** at P62 (3τ = 61.9 ± 12.3; curve-fit *R* = 0.89; *p* < 0.0001), somatosensory **(D)** at P61 (3τ = P61.221.4; curve-fit *R* = 0.82; *p* < 0.0001), and frontal **(G)** at P49 (3τ = P49.1 ± 11.8; curve-fit *R* = 0.83; *p* < 0.0001). Gephyrin expression reached a peak in visual **(B)** at P29 (Peak = 29.3 ± 5.1; curve-fit *R* = 0.61 *p* < 0.0001), somatosensory **(E)** at P13 (Peak = 12.8 ± 5.1; curve-fit *R* = 0.48 *p* < 0.005), and frontal **(H)** at P20 (Peak = 20.4 ± 1.1; curve-fit *R* = 0.60 *p* < 0.0001). Post-synaptic index reached adult levels in visual **(C)** at P25 ( curve-fit *R* = 0.87; *p* < 0.0001, Adult Level = P24.6 ± 7.0), somatosensory **(F)** at P21 (curve-fit *R* = 0.98;* p* < 0.0001; Adult Level = P21.4 ± 2.2), and frontal **(I)** at P30 (curve-fit *R* = 0.95; *p* < 0.0001; Adult Level = P30.2 ± 4.9).

**FIGURE 4:**
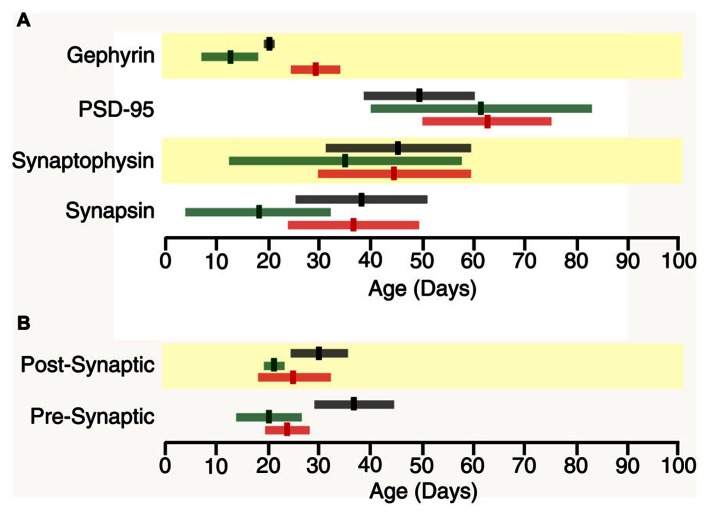
**Time lines for maturation of protein expression and pre- and post-synaptic indices in frontal (black), somatosensory (green) and visual cortex (red) showing the age when adult levels (3τ) or peak in expression were reached (bright bars) and the standard error (light bars) around the 3τs or peak (light bars) (A) Adult levels of synapsin expression overlapped among the three cortical areas (Visual, 3τ = P37.2 ± 12.6; somatosensory cortex, 3τ = P19.0 ± 14.9; Frontal, 3τ = P37.6 ± 13.5).** Adult levels of synaptophysin expression overlapped among the three cortical areas (Visual, 3τ = P44.8 ± 15.1; somatosensory, 3τ = P34.7 ± 22.2; Frontal, 3τ = P45.4 ± 14.2). Adult levels of PSD-95 expression overlapped among the three cortical areas (Visual, 3τ = 61.9 ± 12.3; somatosensory, 3τ = 61.2 ± 21.4; Frontal, 3τ = 49.1 ± 11.8). Peak in gephyrin expression was significantly later in Visual (Peak = 29.3 ± 5.1), than somatosensory (Peak = 12.8 ± 5.1, *p* < 0.05) and Frontal (Peak = 20.4 ± 1.1, *p* < 0.05) cortex. **(B)** The pre-synaptic index reached adult levels earlier in the sensory areas (Visual, 3τ = P23.5 ± 4.2, *p* = 0.06; somatosensory, 3τ = P20.2 ± 6.0, *p* < 0.05) than in frontal cortex (3τ = P36.7 ± 7.8). The post-synaptic index reached adult levels earlier in the sensory areas (Visual, 3τ = 46.4 ± 9.5, *p* < 0.05; somatosensory, 3τ = 43.5 ± 6.1, *p* < 0.005) than in frontal cortex (3τ = 75.6 ± 11.2).

We found substantial increases in PSD-95 expression during development of all three cortical areas, starting from very little expression at P0–P4 to reach adult levels between P49 and P62. In visual cortex, there was a 10-fold increase in PSD-95 expression (**Figure 3A**, curve-fit *R* = 0.89; *p* < 0.0001) between P11 and P62 when adult levels were reached (3τ = P61.9 ± 12.3). Expression of PSD-95 also rose substantially in somatosensory cortex with an eightfold increase in expression between P7 and P61 (3τ = P61.2 ± 21.4; **Figure 3D**, curve-fit *R* = 0.82; *p* < 0.0001). In frontal cortex, there was a fivefold increase in PSD-95 expression (**Figure 3G**, curve-fit *R* = 0.83 *p* < 0.0001) between P11 and P49 (3τ = *p* 49.1 ± 11.8). There were no significant (*z*-test) differences among the three cortical areas in the age when adult levels were reached for PSD-95 expression (**Figure 4A**).

The postnatal development of gephyrin expression followed a different trajectory from the other synaptic proteins, with a bump in expression that was similar to earlier reports counting inhibitory synapses in developing visual cortex (Blue and Parnavelas, 1983). Gephyrin expression levels were similar early (P0, P4) and late(>P60) in development, but we noticed that each cortical area went through an intermediate period when gephyrin expression was elevated (**Figure 3**). The development of gephyrin expression in frontal cortex was well fit by the peak function (curve-fit *R* = 0.60; *p* < 0.0001; **Figure 3H**), showing a twofold bump in expression around P20 (peak P20.4 ± 1.1; **Figure 3**). Since gephyrin expression appeared elevated during that intermediate period in the other cortical areas, and Blue and Parnavelas (1983) had found a transient increase in inhibitory synapses we fit the same peak function to visual and somatosensory cortex and found that the bump was more restricted in time. In visual cortex, the peak occurred at P29 (curve-fit *R* = 0.61; *p* < 0.0001, peak P29.3 ± 5.1; **Figure 3B**), significantly later than the peak in frontal cortex (*z*-test, *p* < 0.05; **Figure 4A**). In contrast, the peak in somatosensory cortex occurred at P13 (curve-fit *R* = 0.48; *p* < 0.005, peak P12.8 ± 5.1; **Figure 3E**), which was significantly earlier than the peak in visual cortex (*z*-test, *p* < 0.05; **Figure 4A**). The brief elevations in gephyrin expression in visual and somatosensory cortex indicate that additional studies with more samples targeted around the developmental ages we have identified, will be needed to obtain more precise estimates of the magnitude and timing of the gephyrin bump.

### THE PSD-95:GEPHYRIN BALANCE SHIFTS FROM GEPHYRIN TO ROUGHLY EQUAL WITH PSD-95

The excitatory orinhibitory balance is a key component of plasticity in the visual cortex (Maffei and Turrigiano, 2008; Morishita and Hensch, 2008). To examine development of the mechanisms involved in regulating the E-I balance we calculated an index of PSD-95:gephyrin expression in visual, somatosensory, and frontal cortex. Interactions between PSD-95 and gephyrin regulate the number of excitatory and inhibitory synapses that affect the physiological E/I balance (Prange et al., 2004; Lardi-Studler et al., 2007; Keith and El-Husseini, 2008). This post-synaptic index is calculated from the difference in expression of PSD-95 and gephyrin divided by the sum (post-synaptic index = (PSD-95 – gephyrin)/(PSD-95 + gephyrin) and can vary between -1 (only gephyrin) and +1 (only PSD-95). We found significant developmental changes in the post-synaptic index for all three cortical areas with a progressive shift from much more gephyrin to balanced expression or slightly more PSD-95 (**Figure 3**). In visual cortex, the shift to a balanced post-synaptic index was reached at P25 (**Figure 3C**, curve-fit *R* = 0.87; *p* < 0.0001, adult level = P24.6 ± 7.0). In somatosensory cortex, the shift to slightly more PSD-95 expression was mature at P21 (**Figure 3F**, curve-fit *R* = 0.98; *p* < 0.0001; adult level = P21.4 ± 2.2). In frontal cortex, the shift in the post-synaptic index was a bit slower and reached adult levels at P30 (**Figure 3I**, curve-fit *R* = 0.95; *p* < 0.0001; adult level = P30.2 ± 4.9). Thus, maturation of the post-synaptic index was similar in the three cortical areas, although there was a trend for frontal cortex to mature later than somatosensory cortex (*z*-test, *p* = 0.051; **Figure 4B**).

### PRINCIPAL COMPONENT ANALYSIS HIGHLIGHTS SIMILARITIES AND DIFFERENCES IN DEVELOPMENT AMONG THE CORTICAL AREAS

**FIGURE 5:**
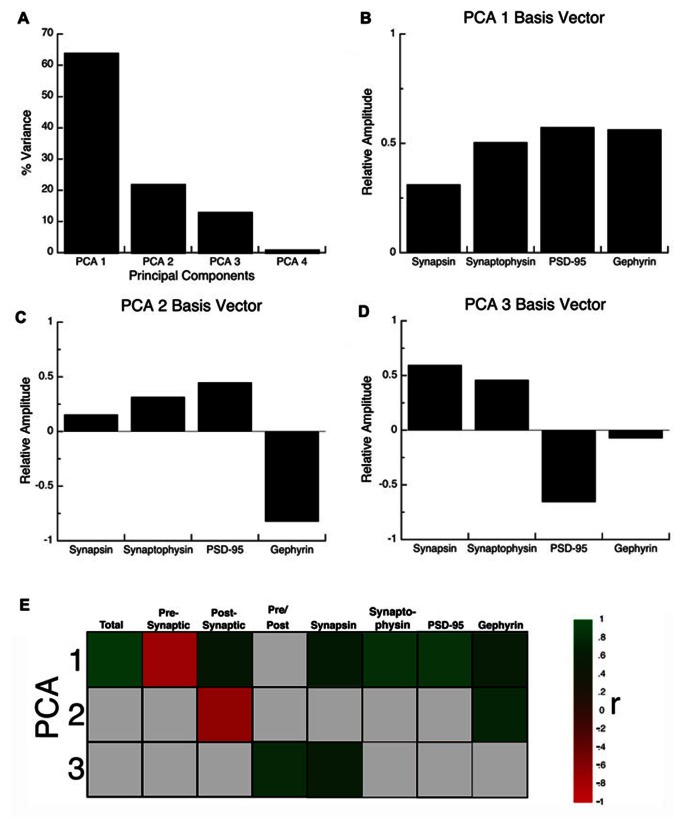
**Principal component analysis. (A)** The percent variance captured by each component of the SVD analysis of protein expression in rat visual, somatosensory, and frontal cortex. The first three principal components represent the significant portion (99%) of the SVD. **(B–D)** The influence of each protein on the three principal components was reflected by the relative amplitude in the basis vectors. **(E)** Significant correlations (colored cells) between the three principal components and the combinations of proteins derived from the basis vectors. The color indicates the magnitude (represented by color intensity) and direction (green indicates positive, red indicates negative) of significant correlations (Bonferroni corrected, *p* < 0.0024).

The synaptic proteome is a complex functional system. To address the multidimensional nature of the combined development of the four proteins in visual, somatosensory, and frontal cortex, we used a data-driven approach and analyzed all of the protein expression using SVD. This allowed us to quantify the underlying principal components that explain this multidimensional data-set. The SVD showed that there were four principal components, the first principal component (PCA 1) explained the greatest proportion of the variance (64%), the second (PCA 2) explained 22% of the variance, the third (PCA 3) explained 13%, and the fourth (PCA 4) explained 1% (**Figure 5A**). We ran a Monte Carlo simulation to determine which components accounted for a significant proportion of the residual variance in the data. The first three components (PCA 1, *p* < 0.0001; PCA 2, *p* < 0.005; PCA 3, *p* < 0.0001) each accounted for a significant proportion and were used for the subsequent analyses.

Each principal component represents a linear combination of the expression of the four proteins and the influence that each protein had on a principal component was reflected by the relative amplitude of the basis vector (**Figure 5**). Analyzing the basis vectors was an important, two-step process, that we used to link the principal components with relevant biological mechanisms (Beston et al., 2010). First, we computed the basis vectors; this provided insights regarding the biological mechanisms driving the data. The basis vectors for PCA 1 (**Figure 5B**) showed positive contributions from all four of the proteins, indicating that it is driven by total expression of synapsin, synaptophysin, PSD-95, and gephyrin. For PCA 2 (**Figure 5C**) the basis vectors showed opposite directions for PSD-95 and gephyrin, the markers for excitatory and inhibitory synapses, respectively, suggesting that this component is linked with the E/I balance. Finally, for PCA 3 (**Figure 5D**) the basis vectors had opposite directions for the pre- versus post-synaptic proteins suggesting that it reflects differences in pre- versus post-synaptic development.

Second, we calculated a set of correlations between the three significant principal components and the four proteins, two indices, and two new measures identified by step one (**Figure 7A**, total protein expression; **Figure 7B**, pre-synaptic:post-synaptic index). To account for multiple comparisons, we performed a Bonferroni correction, and then displayed significant correlations between the 3 principal components and 8 measures (**Figure 5E**; green and red squares, *p* < 0.0021; **Table 2**). The pattern of correlations provided information that described the biological links for each principal component (**Figure 5E**; **Table 2**). The first principal component was characterized by total protein expression (*R* = 0.9913, *p* < 0.0001). The second principal component captured changes in gephyrin expression (*R* = 0.7594, *p* < 0.0001), and the post-synaptic index (E/I balance; *R* = -0.6669, *p* < 0.0001). The third principal component captured differences between pre- versus post-synaptic development (*R* = 0.8034, *p* < 0.0001), and synapsin expression (*R* = 0.6911, *p* < 0.0001).

**Table 2:** principal component analysis correlation.

	**Total Protein**	**Pre-Synaptic**	Post-Synaptic	**Pre/ Post**	**Synapsin**	**Synaptophysin**	**PSD-95**	**Gephyrin**
***r*-value**
PC A 1	0.9913	-0.7663	0.6713	0.2004	0.6992	0.8845	0.8931	0.6449
PC A 2	0.0755	0.3125	-0.6669	-0.4472	-0.0238	-0.2153	-0.3181	0.7594
PC A 3	0.1071	0.1135	-0.0548	0.8034	0.6911	0.3901	-0.3164	-0.0859
***p*-value (*p* < 0.0021)**
PC A 1	0.0000	0.0000	0.0000	0.2149	0.0000	0.0000	0.0000	0.0000
PC A 2	0.6435	0.0496	0.0000	0.0038	0.8841	0.1822	0.0455	0.0000
PC A 3	0.5107	0.4857	0.7370	0.0000	0.0000	0.0128	0.0467	0.5983

**FIGURE 6:**
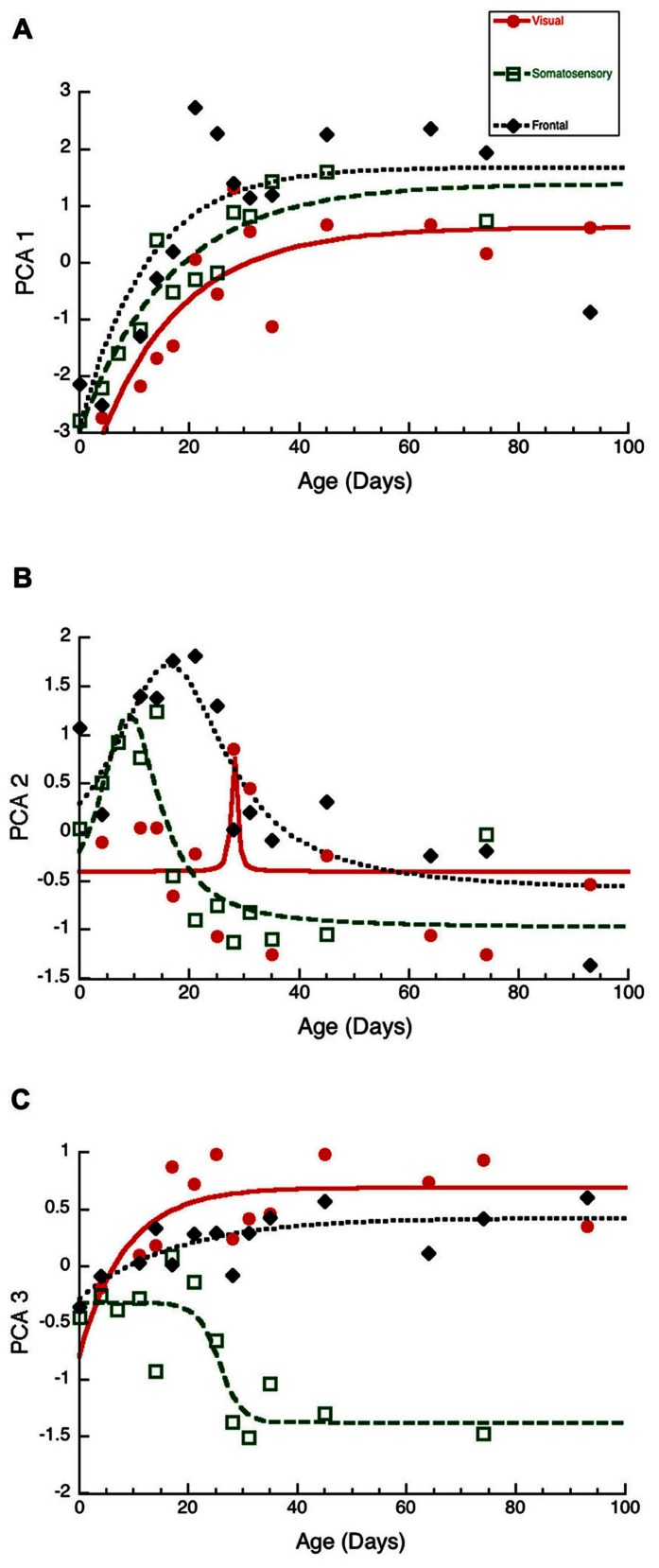
**Developmental changes in the three principal components in visual (red dots, solid line), somatosensory (green squares, dashed lines), and frontal (black diamonds, dotted line) cortex. (A)** Principal component 1. Exponential decay functions were fit to the data for each areas, adult levels are defined as 3τ. Principal component one reached adult levels in visual at P45 (3τ = 44.7 ± 18.3; *R* = 0.85, *p* < 0.0001), somatosensory at P50 (3τ = 49.6 ± 14.8; *R* = 0.94, *p* < 0.0001), and frontal at P37 (3τ = 36.5 ± 19.0; *R* = 0.78, *p* < 0.001). (B) Principal component 2. Peak functions were fit to the data, and the timing of the peak was determined. Principal component two reached a peak in visual at P28 (Peak = 28.1 ± 0.6; *R* = 0.55, *p* < 0.05), somatosensory at P9 (Peak = 9.0 ± 1.2; *R* = 0.86, *p* < 0.0001), and frontal at P16 (Peak = 16.3 ± 1.9; R0.87, *p* < 0.0001). **(C)** Principal component 3. Exponential decay functions were fit to the data for visual, and frontal cortex, and adult levels were defined as 3τ. A sigmoid function was fit to the data for somatosensory cortex, and the timing of the inflection point was determined. Principal component three reached adult levels in visual at P26 (3τ = 25.5 ± 16.8; *R* = 0.69, *p* < 0.01), and frontal at P53 (3τ = 52.5 ± 28.2; *R* = 0.78, *p* < 0.001). The inflection point in somatosensory cortex occurred at P26 (Inflection Point = 25.7 ± 2.1; *R* = 0.87, *p* < 0.0001).

**FIGURE 7:**
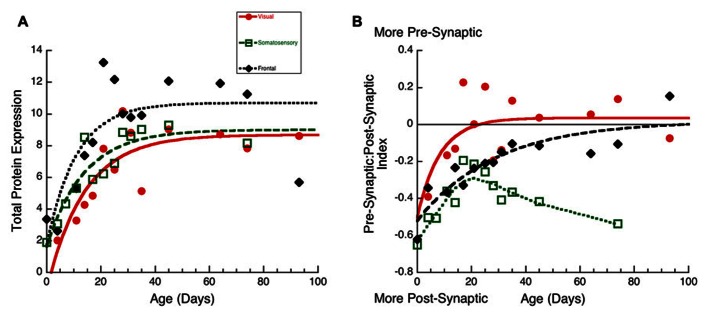
**Developmental changes in total protein expression (A) and the pre-synaptic:post-synaptic Index in visual (red dots, solid line), somatosensory (green squares, dashed lines), and frontal (black diamonds, dotted line) cortex. (A)** Exponential decay functions were fit to the data for each area, adult levels were defined as 3τ. Total protein expression reached adult levels in visual at P42 (3τ = 42.0 ± 17.6; *R* = 0.84, *p* < 0.0001), in somatosensory at P42(3τ = 41.6 ± 13.8; *R* = 0.92, *p* < 0.0001), and in frontal cortex at P33 (3τ = 32.9 ± 17.8; *R* = 0.77, *p* < 0.001). **(B)** Exponential decay functions were fit to the pre-synaptic:post-synaptic index in visual and frontal cortex, adult levels were defined as 3τ. Pre-synaptic:post-synaptic index reached adult levels in visual at P25 (3τ = 25.0 ± 15.0; *R* = 0.64, *p* < 0.05), and in frontal cortex at P86 (3τ = 86.2 ± 35.0; *R* = 0.89, *p* < 0.0001). A weighted average curve was fit to somatosensory cortex.

To visualize the developmental changes for each principal component in the three cortical areas we plotted each component as a function of age (**Figure 6**). The first principal component was strongly correlated with total protein expression (*R* = 0.9913, *p* < 0.0001) and showed a progressive increase in the three cortical areas. The development of PCA 1 was well fit by tau decay functions that had overlapping developmental trajectories among the three cortical areas (visual, 3τ = 44.7 ± 18.3; *R* = 0.85, *p* < 0.0001; somatosensory, 3τ = 49.6 ± 14.8; *R* = 0.94, *p* < 0.0001; frontal, 3τ = 36.5 ± 19.0; *R* = 0.78, *p* < 0.001). We found no significant differences between the cortical areas for the age when PCA 1 reached adult-levels (*z*-test, n.s). This suggested that developmental increases in expression of these four synaptic proteins occurred in unison across these cortical areas. We then plotted the development of total protein expression (**Figure 7A**) and found that adult-levels were reached at similar ages (P33-P42; *z*-test, n.s) in the three cortical areas (visual, 3τ = 42.0 ± 17.6; *R* = 0.84, *p* < 0.0001; somatosensory, 3τ = 41.6 ± 13.8; *R* = 0.92, *p* < 0.0001; frontal, 3τ = 32.9 ± 17.8; *R* = 0.77, *p* < 0.001)

The second principal component (**Figure 6B**) captured changes in the expression of both gephyrin (*R* = 0.7594, *p* < 0.0001) and the post-synaptic index (E/I balance; *R* = 0.7594, *p* < 0.0001). The early phase (P0-P30) of PCA 2 development had a peak in each area. The peak occurred first in somatosensory (Peak = 9.0 ± 1.2; *R* = 0.86, *p* < 0.0001), second in fontal (Peak = 16.3 ± 1.9; *R* = 0.87, *p* < 0.0001), and third in visual cortex (Peak = 28.1 ± 0.6; *R* = 0.55, *p* < 0.05). The timing of the peak was significantly earlier in somatosensory versus frontal cortex (*z*-test, *p* < 0.0001), and frontal versus visual cortex (*z*-test, *p* < 0.0001). The later phase (>P30) of PCA 2 had a separation between somatosensory cortex and the other areas, reflecting the shift in the E/I balance to relatively more PSD-95 in somatosensory cortex (**Figure 3F**). The PCA 2 results suggest a two stage developmental process involving an early bump in gephyrin occurring at slightly different ages in these areas (somatosensory, P9; frontal, P16; visual, P28), and a prolonged shift in the E/I balance that is greater in somatosensory cortex.

The third principal component (**Figure 6C**) was correlated with the differences between pre- and post-synaptic proteins (*R* = 0.8034, *p* < 0.0001) and Synapsin (*R* = 0.6911, *p* < 0.0001). The development of PCA 3 expression started at a similar level for the three areas but then diverged by P20, with somatosensory cortex taking a different trajectory from either visual or frontal cortex (**Figure 6C**). The development of PCA 3 in visual and frontal cortex was well fit by tau decay functions with adult levels (3τ) reached at P26 in visual cortex (3τ = 25.5 ± 16.8; *R* = 0.69, *p* < 0.01) and at P53 in frontal cortex (3τ = 52.5 ± 28.2; *R* = 0.78, *p* < 0.001). These ages, however, were not significantly different because of variability in visual and frontal cortex. PCA 3 for somatosensory cortex turned away from the other areas at about P20, reached the falling inflection point at P26 (Inflection Point = 25.7 ± 2.1; *R* = 0.87, *p* < 0.0001), and the minimum at about P30. The final level of PCA 3 in somatosensory cortex was different from either visual (Tukey’s, *p* < 0.0001) or frontal cortex (Tukey’s, *p* < 0.0001). We plotted the index of pre-synaptic:post-synaptic difference in protein expression to visualize the developmental changes in this measure (**Figure 7B**). Both visual and frontal cortex developed to reach roughly equivalent expression of the pre- and post-synaptic proteins, and there was a trend (*z*-test, *p* = 0.055) toward later maturation in frontal cortex. In contrast, the mature somatosensory cortex had much greater expression of post-synaptic proteins (**Figure 7B**). These analyses of PCA 3 and the pre-synaptic:post-synaptic index uncovered a divergence in development of the synaptic proteome in somatosensory cortex.

## DISCUSSION

### RESULTS SUPPORT AN INTEGRATED NETWORK OF DEVELOPMENT

By measuring a set of pre- and post-synaptic proteins and using a neuroinformatics approach to analyze expression levels we found that the pattern of maturation reflects three underlying components with clear links to the development of individual proteins and the balances between sets of synaptic proteins. First, an integrated network among the three cortical areas describes the largest part of development. We found substantial overlap among the three cortical areas in the timing of development for both individual proteins, the post-synaptic index, and the total expression of the four synaptic proteins (PCA 1). Furthermore, the PCA showed that the overlapping trajectories among visual, somatosensory and frontal cortex accounted for 64% of the variance in development of the synaptic proteins. Second, each cortical area had an early wave of development linked with a transient bump in gephyrin expression and the later tail that was the gradual emergence of an E-I between PSD-95 and gephyrin expression (PCA 2). The wave in frontal cortex overlapped with both somatosensory and visual cortex, so there was not an obvious hierarchical sequence of development among the cortical areas. We did, however, find that the wave was broader in frontal cortex and there was significantly later maturation of the pre-synaptic index, which together support later maturation of frontal cortex. Third, somatosensory cortex diverged from both visual and frontal cortex during maturation of the balance between pre- and post-synaptic proteins (PCA 3). Somatosensory cortex was strongly dominated by the expression of the post-synaptic proteins, while visual and frontal cortex reached a balance. Together, our results show that multiple factors, dominated by largely overlapping trajectories, underlie development of these pre- and post-synaptic proteins in rat visual, somatosensory, and frontal cortex.

In this study we quantified levels of expression of synaptic proteins in visual, somatosensory and frontal cortex as a way to assess synaptic development across the cortex. We used this measure because the accumulation of pre- and post-synaptic proteins is an important part of the process of synaptogenesis that is initiated by intracellular signals triggered by contact between axons and dendrites (McAllister, 2007). Other aspects of synapse formation, such as when and where synapses form, how proteins accumulate at the synapse, and the location of proteins at or near nascent synapses, are not captured by simply quantifying protein expression. These questions are better addressed with anatomical, physiological, and imaging techniques that get at synapse structure, function, and dynamics. For example, imaging studies have shown that during synapse formation PSD-95 and gephyrin are extremely motile in the dendritic shaft and that motility decreases with development (Gray et al., 2006; Dobie and Craig, 2011). But we do not know how similar the degree of motility is in various cortical areas and if it does vary that would impact synapse development and consolidation.

### PRE-SYNAPTIC DEVELOPMENT

The rate of development of the pre-synaptic proteins, synapsin and synaptophysin, was similar among the cortical areas providing support for an integrated network of pre-synaptic development. The balances between synapsin and synaptophysin is tied to the potential for functional synaptic vesicle cycling, since low levels of synapsin and synaptophysin reduce pre-synaptic functioning and transmitter release (Hopf et al., 2002). We are the first to quantify development of the balance between synapsin and synaptophysin expression and found very tight developmental trajectories in each cortical area. Initially, there was more synapsin expression in rat cortex, followed by a rapid increase in synaptophysin until balanced or slightly more synaptophysin expression was reached. Interestingly, the pre-synaptic balance matured later in frontal cortex suggesting slower functional development. Synapsin is found at all glutamatergic and GABAergic synapses but synaptophysin expression is low levels at GABAergic synapses (Gronborg et al., 2010; Micheva et al., 2010). The early expression of synapsin may reflect earlier development of GABAergic synapses in rat cortex (Ben-Ari et al., 2007) and be linked with our finding of early expression of gephyrin. Synapsin maintains the abundance and organization of vesicles at the pre-synaptic terminal (Bykhovskaia, 2011), and the phosphorylation state regulates the pool of vesicles available for exocytosis (Bahler et al., 1990). Synaptophysin interacts with synaptobrevin to regulate synaptic vesicle retrieval and endocytosis (Gordon et al., 2011). Development of the pre-synaptic balance suggests early maturation of vesicle exocytosis mechanisms, with a slight lag in development of endocytosis mechanisms. This balance is likely an important mechanism affecting vesicle cycling and contributing to activity-dependent processes that rely on the physiological maturation of neuronal responsiveness from weak and sluggish, to strong and sustained firing patterns needed for efficient synaptic transmission (Rust et al., 2002; Lu et al., 2006).

### POST-SYNAPTIC DEVELOPMENT

On the post-synaptic side, we examined expression of PSD-95 and gephyrin, the scaffolding proteins for GluA/GluN and GABA_A_ receptors, respectively. We found substantial overlap among the three cortical areas in the rate of development of PSD-95, while the peaks for gephyrin expression progressed from somatosensory to frontal to visual cortex. The post-synaptic balance between PSD-95 and gephyrin followed tight developmental trajectories in each area shifting from much more gephyrin and then an increase in PSD-95 to reach balanced expression. The time course of this shift was similar among the three areas, further supporting an integrated network of development. The PSD-95:Gephyrin balance provides an indication of the relative number of excitatory versus inhibitory synapses that will contribute to the physiological E-I balance. Importantly, interactions between these two scaffolding proteins influence both the relative number of excitatory and inhibitory synapses and the physiological E-I balance (Keith and El-Husseini, 2008). Over expression of gephyrin clusters during synapse formation causes a reduction in PSD-95 expression without reducing the total number of synapses, suggesting that gephyrin interacts with PSD-95 to control the balance between excitatory and inhibitory synapses (Lardi-Studler et al., 2007). In addition, PSD-95 over expression enhances the size of excitatory synapses and reduces the number of inhibitory contacts leading to a physiological shift in the E-I balance (Prange et al., 2004). Very early in development of the visual cortex, the physiological E-I balance favors excitation, then an increase in inhibition shifts the balance to trigger the onset of the critical period (Maffei and Turrigiano, 2008; Morishita and Hensch, 2008). Our finding of more gephyrin early on might seem counter to those physiological findings, but initially GABA_A_ receptors are depolarizing due to an abundance of the immature chloride co-transporter NKCC1. The developmental switch to the mature chloride co-transporters, KCC2, causes hyperpolarization upon activation of GABA_A_ receptors (Ben-Ari, 2002), and the hyperpolarizing effect of GABA_A_ receptors becomes apparent in visual cortex at P10 (Ikeda et al., 2003). Thus, our finding of early expression of gephyrin relative to PSD-95 means that when KCC2 is expressed there can be a very rapid switch from excitation to inhibition, that could re-set the physiological E-I balance and trigger the critical period.

### EMERGENCE OF SYNAPTOPHYSIN AND PSD-95 EXPRESSION COINCIDE WITH THE SWITCH TO FUNCTIONAL SYNAPSES

Using a small number of synaptic proteins our study complements and extends previous work on synaptic development in rat cortex by providing a rich picture of the developmental changes in these building blocks for synapses. Analysis of the total expression of the synaptic proteins showed highly overlapping development among the cortical areas. Moreover, the links between the set of proteins and synaptic function suggests that these developmental trajectories will be helpful as a framework for connecting with physiological results and guiding future studies of synaptic development. For example, a significant event in synaptic development is the switch from silent to functional synapse and it requires the formation of both pre- and post-synaptic components (Isaac et al., 1995; Liao et al., 1995; Durand et al., 1996). That switch occurs at P9-11 in visual (Rumpel et al., 1998) and P8-9 in somatosensory cortex (Isaac et al., 1997), ages that are similar to when we found the onset of synaptophysin and PSD-95 expression. Furthermore, maturation of pre-synaptic transmission is required for the switch from silent to functional synapses (Renger et al., 2001), suggesting that appropriate levels of synapsin and synaptophysin are needed to support vesicle exo- and endocytosis to sustain neural activity. In addition, PSD-95 plays a critical role in the conversion of synapses from silent to functional, since PSD-95 knock-out mice have an increased proportion of silent synapses (Beique et al., 2006). Surprisingly, those silent synapses are located on morphologically mature spines, showing that the anatomical presence of a mature spine is not indicative of a functional synapse (Beique et al., 2006). Perhaps the balance between pre- and post-synaptic proteins will become a helpful tool to complement physiological studies of the emergence of functional synapses. For example, development of the balance between pre- and post-synaptic proteins took a different trajectory in somatosensory cortex from either visual or frontal cortex. At about P20, PCA 3 progressed toward relatively more pre-synaptic expression in visual and frontal cortex, but in somatosensory cortex it shifted to more post-synaptic expression. That shift in PCA3 could be due in part to the decrease in pre-synaptic release probability that occurs in somatosensory cortex between P12-28, but does not happen in visual cortex (Cheetham and Fox, 2010).

### INCREASED PROTEIN EXPRESSION IS ASSOCIATED WITH SYNAPTIC STABILIZATION

The synaptic proteins that we quantified are some of the most commonly used markers in imaging studies to visualize the dynamic nature of developing synapses. Imaging to track vesicle turnover has shown that synapsin and synaptophysin are required to stabilize the position and efficacy of pre-synaptic boutons (Hopf et al., 2002). This raises the possibility that the rapid development of a balance between these pre-synaptic proteins contributes to early stabilization of pre-synaptic function and underlies differences in the maturation of pre-synaptic function in somatosensory and visual areas (Cheetham and Fox, 2010). On the post-synaptic side, there is a dynamic pool of PSD-95 shared by synapses within a dendrite. This motility is especially high during early stages of cortical synaptic development when PSD-95 turnover is rapid as it diffuses among neighboring spines (Gray et al., 2006). The rapid turnover is greatest during the critical period (P10-21 in barrel cortex of mice) and coincides with the time when we found that PSD-95 expression was developing rapidly. Motility of PSD-95 slows substantially by P100, and larger synaptic boutons retain PSD-95 for longer, suggesting that changes in the kinetics of PSD-95 diffusion contribute to synapse stability (Gray et al., 2006). The greatest changes in development of PSD-95 overlapped with the stages when imaging studies found the greatest dynamics for the pool of PSD-95. Furthermore, PSD-95 expression matured at about P60 in visual and somatosensory cortex, and about P50 in frontal cortex, ages that coincide with slowing of PSD-95 turnover to its constituent level and increased synapse-specific retention times (Gray et al., 2006). Together, these results suggest that the expression level of PSD-95 during development reflects the dynamic pool of PSD-95 that diffuses in dendrites among developing synapses.

The prolonged development of PSD-95 points to a long period when excitatory synapses are stabilized in the cortex. In contrast, we have previously found more rapid emergence of AMPA receptors (GluR2 and pGluR2) in rat visual and frontal cortex (Murphy et al., 2012). The time course of AMPA receptor development was similar to the timing found in this study for the pre-synaptic proteins. These results provide support for both parallel and hierarchical development of synapses (McAllister, 2007) where certain pre- (e.g., synapsin and synaptophysin) and post-synaptic (e.g., AMPA receptors) follow similar time courses, while other components (e.g., PSD-95) develop later. Perhaps this reflects the initial emergence of functional synapses, followed by experience-dependent fine tuning and stabilization of synapses.

The GABAergic synapse scaffolding protein gephyrin also has significant motility, on the order of seconds to hours (Dobie and Craig, 2011). Furthermore, that motility is negatively correlated with the density of gephyrin clusters (Kuriu et al., 2012), so that more gephyrin equates to less motility. In the current study, we found that gephyrin development in rat cortex goes through a brief period of increased expression similar to the period of increased density of GABAergic (type II) synapses in rat visual cortex (Blue and Parnavelas, 1983). Perhaps the bump in gephyrin expression imparts a period of reduced gephyrin motility that heightens stabilization of GABAergic synapses. Interestingly, gephyrin expression also has a bump in expression during development of human visual cortex, but it is more prolonged and extends until late childhood (Pinto et al., 2010).

The timing of the gephyrin bump coincides with the peak of the critical period in visual and somatosensory cortex, suggesting that it is part of the process that facilitates critical period experience-dependent plasticity. For example, the rapid ocular dominance changes caused by monocular deprivation depend on an increase in inhibition (Hensch and Fagiolini, 2005). In frontal cortex, the broader and shallower gephyrin bump may reflect differences in the magnitude and duration of experience-dependent plasticity between sensory and non-sensory areas that have been characterized by the latent impact of maternal separation during development on stress responses later in life (Meaney, 2001; Monroy et al., 2010). The bump in gephyrin expression may turn out to be a helpful measure for characterizing the strength and length of critical periods in the cortex.

This study has shown that there is substantial overlap among cortical areas in development of synaptic proteins and it provides a simple framework for future studies that aim to detail development of the cortical connectome. The developmental trajectories for the four synaptic proteins, and the balances among them, highlight important milestones in development that are coincident with the emergence of functional synapses, and periods of increased synaptic dynamics. Importantly, the pre- and post-synaptic indices link with both synaptic function and the E-I balance. We anticipate that these trajectories can help focus time consuming anatomical and imaging studies on critical ages in synaptic development.

## Conflict of Interest Statement

The authors declare that the research was conducted in the absence of any commercial or financial relationships that could be construed as a potential conflict of interest.
